# Supplementary material for: A Bioinformatic Study of Genetics Involved in Determining Mild Traumatic Brain Injury Severity and Recovery
Source: Biomedicines. 2025 Oct 30;13(11):2669. doi: 10.3390/biomedicines13112669 (PMC12650054; doi:10.3390/biomedicines13112669)
Supplement: Supplementary file 1 [file biomedicines-13-02669-s001.zip › Supplementary File SA.pdf]

<u>Supplementary file SA</u>:

RNA sequencing analysis and Linux commands and R scripts

RNA sequencing data analysis:

Raw data from the GSE123336 dataset was downloaded from the GEO repository. After trimming the fastq files and removing adapters, BWA aligned fastq files with the reference genome GRCh38 (hg38) and mature miRNA sequences. GRCh38 was downloaded from the UCSC website (https://genome.ucsc.edu/) and mature miRNAs were downloaded from the miRBase database version 22.

1- Alignment with BWA:

The first step was to create a reference index for both genome and mature miRNA sequences.

# bwa index

bwa index '/Path directory/hg38.fa'

bwa index '/Path directory/mature.fa'

# aligning single end reads

*bwa aln ref.fa *.fastq > *.sai*

bwa aln /Path directory/hg38.fa '/Path directory/SRR8280405_trimmed.fastq' > '/Path directory/SRR8280405.sai'

bwa aln /Path directory/mature_fa/mature.fa '/ Path directory/mature_fa /SRR8280405-trimmed.fastq' > '/ Path directory/mature_fa /SRR8280405.sai'

The second step was generating SAM files and BAM files.

# bwa same

bwa same index file file.sai. fa > .sam*

bwa samse '/Path directory/hg38.fa ' '/Path directory/SRR8280405.sai' '/Path directory/SRR8280405_trimmed.fastq' > '/Path directory/SRR8280405_trimmed.sam'
bwa samse '/Path directory/mature_fa/mature.fa' '/Path directory/ SRR8280405.sai' '/Path diretory/SRR8280633.trimmed.fastq' > '/Path directory/ SRR8280405.trimmed.sam'

# Samtools

samtools view -b '/Path directory/SRR8280405_trimmed.sam' > '/Path directory/SRR8280405_trimmed.bam'

```
samtools view -b '/Path directory/mature_fa/SRR8280405.trimmed.sam' > '/Path directory/mature_fa/SRR8280405.trimmed.bam'

samtools sort '/Path directory/SRR8280405_trimmed.bam' -o '/Path directory/SRR8280405.sorted'

samtools sort '/Path directory/mature_fa/SRR8280405.trimmed.bam' -o '/Path directory/mature_fa/SRR8280405.sorted'
```

The last step used FeatureCounts to create a count matrix from the sorted files.

# FeaturCounts

```
./featureCounts -t miRNA -F GTF -g ID -a '/Path directory/hsa.gff3' -o '/Path directory/FeatureCountsTable.txt' '/Path directory/SRR8280405.sorted'

./featureCounts -t miRNA -F GTF -g ID -a '/Path directory/mature_fa/mature.fa' -o '/Path directory/mature_fa/FeatureCountsTable.txt' '/Path directory/mature_fa/SRR8280405.sorted'
```

The count matrix was obtained by aligning reads with the reference genome and mature miRNA sequences were merged for differential expression miRNA analysis.

2- Differential expression analysis with DESeq2

Differential expression analysis of the miRNA-seq counts was done using DESeq2 package in R studio software. For the DESeq2 package, miRNA counts and their grouping string were read in.

```
< Library (DESeq2)

< countdata = read.table("Path/FeatureCountsTable.txt" , header = T)

< rownames(countdata) = countdata$Geneid

< countdata = countdata[, -(1:6)]

< colnames(countdata) = gsub(".sorted", "", colnames(countdata))

< sample_key<-read.csv("Path/Pheno2.new.csv")

< sample_key$condition = as.factor(sample_key$condition)

< dds<-DESeqDataSetFromMatrix(countData = countdata, colData = sample_key, - design= ~condition)

< dds

< dds<-DESeq(dds)

< cnt = counts (dds, normalized = T)
```

```
< write.table(cnt,file = "cnt-miRNA.txt", row.names=T, col.names = T, sep="\t", quote = F)
####codition_2_vs_1(0day injury vs Normal group)
< dif = data.frame(results(dds, c("condition", "2", "1")))
< dif$padj = p.adjust(dif$pvalue, method = "BH")
< dif = dif[order(dif$padj),]
< write.table(dif,file = "Path/codition_2_vs_1.txt", row.names=T, sep="\t", quote = F)
< dif$geneID = rownames(dif)
< genes.sig <- subset(dif, padj < 0.05)
< geneID <- unique(genes.sig$geneID)
< write.table(geneID,file = "Path/ genes.sig-name (condition_2_vs_1).txt" , quote = F,
row.names = F, col.names = F)
####codition_3_vs_1(47/48 hours after injury vs Normal group)
< dif2 = data.frame(results(dds, c("condition", "3", "1")))
< dif2$padj = p.adjust(dif2$pvalue, method = "BH")
< dif2 = dif2[order(dif2$padj),]
< write.table(dif,file = "Path/codition_3_vs_1.txt", row.names=T, sep="\t", quote = F)
< dif2$geneID = rownames(dif2)
< genes2.sig <- subset(dif2, padj < 0.05)
< geneID2 <- unique(genes2.sig$geneID)
< write.table(geneID2,file = "Path/genes.sig-name (condition_3_vs_1).txt" , quote = F,
row.names = F, col.names = F)
#####codition_4_vs_1(1 week following injury vs Normal group)
< dif3 = data.frame(results(dds, c("condition", "4", "1")))
< dif3$padj = p.adjust(dif3$pvalue, method = "BH")
< dif3 = dif3[order(dif3$padj),]
< write.table(dif3,file = "Path/codition_4_vs_1.txt", row.names=T, sep="\t", quote = F)
< dif3$geneID = rownames(dif3)
< genes3.sig <- subset(dif3, padj < 0.05)
< geneID3 <- unique(genes3.sig$geneID)
< write.table(geneID3,file = "Path/genes.sig-name (condition_4_vs_1).txt" , quote = F,
row.names = F, col.names = F)
```

Run DESeq2 analysis for different miRNA expression levels based on number of hits to the head (conditions)

```
< setwd ()

< countdata = read.table("FeatureCountsTable.txt" , header = T)

< rownames(countdata) = countdata$Geneid

< countdata = countdata[, -(1:6)]

< colnames(countdata) = gsub(".sorted", "", colnames(countdata))

< sample_key<-read.csv("Pheno2.csv")

< sample_key$condition = as.factor(sample_key$condition)

< dds<-DESeqDataSetFromMatrix(countData = countdata, colData = sample_key, design=
~condition)

< dds

< dds<-DESeq(dds)

< cnt = counts(dds, normalized = T)

< write.table(cnt,file = "cnt2-miRNA.txt", row.names=T, col.names = T, sep="\t", quote = F)

####codition_1_vs_0 (Over 20 hits to the head vs 0)

< dif = data.frame(results(dds, c("condition", "1", "0")))

< dif$padj = p.adjust(dif$pvalue, method = "BH")

< dif = dif[order(dif$padj),]

< write.table(dif,file = "codition_1_vs_0.txt", row.names=T, sep="\t", quote = F)

< dif$geneID = rownames(dif)

< genes.sig <- subset(dif, padj < 0.05)

< geneID <- unique(genes.sig$geneID)

< write.table(geneID,file = "genes.sig-name (condition_1_vs_0).txt" , quote = F, row.names =
F, col.names = F)

####codition_2_vs_0 (Less than 20 hits to the head vs 0)

< dif2 = data.frame(results(dds, c("condition", "2", "0")))

< dif2$padj = p.adjust(dif2$pvalue, method = "BH")

< dif2 = dif2[order(dif2$padj),]

< write.table(dif,file = "codition_2_vs_0.txt", row.names=T, sep="\t", quote = F)

< dif2$geneID = rownames(dif2)
```

```
< genes2.sig <- subset(dif2, padj < 0.05)

< geneID2 <- unique(genes2.sig$geneID)

< write.table(geneID2,file = "genes.sig-name (condition_2_vs_0).txt" , quote = F, row.names = F, col.names = F)

####condition_3_vs_0 (Less than 10 hits to the head vs 0)

< dif2 = data.frame(results(dds, c("condition", "3", "0")))

< dif2$padj = p.adjust(dif2$pvalue, method = "BH")

< dif2 = dif2[order(dif2$padj),]

< write.table(dif,file = "codition_3_vs_0.txt", row.names=T, sep="\t", quote = F)

< dif2$geneID = rownames(dif2)

< genes2.sig <- subset(dif2, padj < 0.05)

< geneID2 <- unique(genes2.sig$geneID)

< write.table(geneID2,file = "genes.sig-name (condition_3_vs_0).txt" , quote = F, row.names = F, col.names = F)
```
