# Supplementary material for: A Bioinformatic Study of Genetics Involved in Determining Mild Traumatic Brain Injury Severity and Recovery
Source: Biomedicines. 2025 Oct 30;13(11):2669. doi: 10.3390/biomedicines13112669 (PMC12650054; doi:10.3390/biomedicines13112669)
Supplement: Supplementary file 1 [file biomedicines-13-02669-s001.zip › Supplementary File SB.pdf]

Supplementary file SB:

Explanation of the 11 mTBI candidate genes identified in our study

Apolipoprotein E allele 4 (APOE e4)

Evidence from recent research indicates that *APOEe4* contributes to poorer recovery after brain injuries [112]. *APOE* is believed to be one of the key genes involved in neuronal cytoskeleton maintenance, neuronal synaptic transmission, neurotic dendrite, and synapse formation [113-116]. Chiang et al. identified a positive genetic link between *APOE* genotypes and brain injury outcomes and found that individuals with the *APOEe4* allele were more likely to have a poor TBI outcome [117]. Whereas other clinical trials have not found a link between the *APOEe4* and a worse result following a TBI, these variations may be related to the severity of injuries or time of sampling post-injury [118,24].

Glial fibrillary acidic protein (GFAP)

GFAP is another important protein found in the central nervous system, found within astrocytes. *GFAP* is an intermediate filament that is regulated by the *GFAP* genes and is regarded as a valuable marker for detecting brain injury. In addition to its apparent connection to mTBI-related alterations, this protein is specific to the central nervous system [119,105] *GFAP* seems to be able to identify TBI and anticipate the necessity for neurosurgical intervention up to seven days after trauma [23]. *GFAP* elevation has been linked to astrocyte and axonal injury, and some studies have shown a significant increase in the level of *GFAP* in the serum of TBI patients [120-122]. Another study established that *GFAP* leaks from damaged cells following TBI, and the amount is related to injury severity and outcome [123] this group also noted that *GFAP* does not increase after non-brain traumas.

Brain derived neurotrophic factor (BDNF)

As shown by hub gene analysis, another important gene associated with mTBI outcomes is *BDNF*. Based on the GO terms, *BDNF* is involved in synapse assembly, cognition, nervous system processes, memory, and the negative regulation of neuronal apoptosis. It has been considered to play a crucial role in the cellular processes that occur during TBI recoveries, such as neuronal survival, axonal sprouting, and synaptogenesis [123,124]. *BDNF* influences the performance of existing synaptic connections as well as the development of new synaptic connections [27]. Therefore, changes in *BDNF* function, whether through synapsis or leak, are thought to influence behaviour [27,125]. Studies show in the heterozygous condition, reduced *BDNF* actually increases body mass and aggressiveness in mice [125]. Furthermore, many studies have found that the *BDNF* polymorphism (val66met) influences cognitive function, neurodegenerative and neuroinflammatory disease following TBI, even though cognitive impairment after brain injury is multifactorial and depends on a variety of factors such as gender, age, severity, and type of brain damage [124,126].

S100-calcium binding protein B (S100B)

A low affinity calcium-binding protein called *S100B-* is a hub gene, produced in glial and Schwann cells, that controls calcium homeostasis in the intracellular space [127-130]. During an astroglial injury, *S100B-* is released from damaged cells into the serum or cerebrospinal fluid (CSF), causing changes in the level of calcium [130]. ***S100B-*** levels have also been shown to

increase as a result of BBB instability [131]. Previous research has shown that a high concentration of S100B- during the early stages of TBI can predict a poor prognosis [22].

Catechol-O-methyltransferase (COMT)

COMT is another important gene determined in this study. This gene encodes an enzyme that plays an essential part in metabolic degradation of catecholamine, dopamine, and norepinephrine called catechol-O-methyltransferase [132]. In accordance with GO, *COMT* is associated with learning, short-term memory, and cognitive function, and the KEGG pathway analysis revealed that *COMT* is one of the main genes in the dopaminergic synapse pathway. The link between *COMT* and cognition is well understood. However, there has not been minimal effort to understand the effect of the *COMT* allele on cognitive function following mTBI. Lipsky et al. found a connection between the *COMT* Val158Met polymorphism and a component of frontal-executive performance after TBI [107]. In this study they realized that *COMT*, which is likely linked with the level of endogenous dopamine, may impact the imbalances of frontal executive performance after TBI [107]. However, further research is required to determine exactly how *COMT* alleles affect cognitive function following a head trauma.

Dopamine receptor D2 (DRD2)

Dopamine (DA) neurotransmission in the healthy brain controls attention, working memory, information processing speed, and cognitive performance [133,81]. These functions are associated with frontal lobe regions that are densely projected with DA-rich striatum [134] and many of the long-term cognitive problems associated with brain damage could be explained by abnormal dopaminergic (DAergic) signaling [80]. One of the main genes involved in the DAergic pathway is *DRD2* which encodes the D2 subtype of the dopamine receptor. Polymorphisms of *DRD2* are quite prevalent and several studies have demonstrated that *DRD2* polymorphisms affect cognitive recovery from TBI [81,135]. One of these SNPs is rs1800497 in the *ANKK1* gene which is known as a *DRD2* "TAQ1 A" allele [136]. The presence of the T-allele in rs1800487 has been linked to a 40% decrease in an expression of the D2 receptor in the striatum and possibly other cortical brain regions [135,136]. McAllister et al. found that individuals with mTBI, who were also T-allele positive, exhibited poor performance on all Continuous Performance Test measures such as the California Verbal Learning Test [135], which aims to assess important elements of cognitive psychology such proactive disruption, serial position effects, repetitive learning, and semantic structure [137].

Calcium voltage-gated channel subunit alpha1 A (CACNA1A)

According to KEGG pathway analysis, *CACNA1A* is another gene related to the dopaminergic synapse pathway. *CACNA1A* encodes the α_{1A} subunit of the neuronal calcium channel [138], and polymorphism of this gene can influence the downstream effects of calcium influx into neurons during brain injury [138,139]. In a small case study, Kors et al., found that a novel C/T substitution mutation at codon 218 in *CACNA1A* caused a serine to leucine switch, which resulted in delayed cerebral edema following mTBI and familial hemiplegic migraine (FHM)[138]. Another study by this group reported early seizures in two patients with mTBI who had the same mutation on the *CACNA1A* gene (serine/leucine) [140]. However, to understand the molecular mechanisms of the *CACNA1A* polymorphism, as well as its impact on mTBI outcomes and treatment, more research is needed.

Aquaprin-4 (AQP4)

Aquaprin-4 (*AQP4*) is the most important water channel in the central nervous system, carrying the majority of water into brain cells while also controlling brain water balance [141]. *AQP4* is mainly expressed in the astrocytic end foot processes near intracerebral vessels and at the ventricular interface [142]. Previous studies have shown that the presence of brain edema after TBI is one of the most important predictors of brain injury outcome [141]. Recent studies have shown that *AQP4* expression is changed significantly in both clinical and trial brain injury, suggesting that genetic variations to these channels may affect the degree of edema [143-146]. In an experimental TBI study, *AQP4* expressions increased in the glia limitans (i.e., the outermost later of nervous tissue, just under the pia mater), but perivascular *AQP4* expression decreased during the early phase when vasogenic edema was present [147]. Dardiotis et al., found that specific variations of the *AQP4* gene were associated with the six-month clinical outcome following TBI [141]. The *AQP4* is involved in brain edema formation following TBI. Consequently, for clinical outcome following mTBI, understanding the role of *AQP4* as well as identifying potential aquaporin modulators would be promising for predicting outcome of brain injury.

Ubiquitin carboxy 3-hydrolase L1 (UCHL1)

One of the most well-studied protein biomarkers associated with brain injury is *UCHL1*. *UCHL1* or neuronal-specific protein gene product 9.5 (PGP9.5) is a highly abundant protein in the brain. Approximately 5–10% of total neuronal protein is made up of this protein [104,148]. In the brain, *UCHL1* is primarily located in neuronal cells, which makes it a promising biomarker of neuronal injury [149]. *UCHL1* is a multifunctional protein that is involved in cell survival, maintenance of axonal integrity, and ubiquitin-proteasome pathway (UPP) control in neurons [150-152]. The *UCHL1* protein is involved in adding or removing ubiquitin from proteins that are about to be degraded by the ATP-dependent proteasome pathway [148,153,154]. Because this system is responsible for removing malformed or dysfunctional proteins, UPP failure could be a factor in aggravating axonal injury after a TBI [148]. *UCHL1* is a stable protein with a low-molecular-weight that is secreted from damaged neurons, it enters the CSF and can then be identified in the systemic circulation [155]. Researchers that examined serum biomarkers >32 hours after an injury discovered that *UCHL1* was elevated in the serum of adults and children with mild to severe TBI and was directly associated to damage severity [156]. Papa et al., reported a significant increase in the level of *UCHL1* following severe brain injury, and that it was detectable in body fluids such as CSF fairly early after injury (at 24, 48, and 72-hours following injury), suggesting that it might be used as a biomarker to help determine the severity of TBI [155].

Myelin Basic Protein (MBP)

The myelin basic protein (*MBP*) gene is another potential biomarker related to brain injury. This gene codes for protein that is a significant component of the myelin sheath of oligodendrocytes of the central nervous system and Schwann cells of the peripheral nervous system [158]. *MBP* can be released into the CSF and serum after the shearing of brain white matter that causes diffuse axonal injuries, and it has been reported to remain increased for up to 2 weeks after the injury [159,160]. *MBP*, through an inflammatory response, can cause the BBB to open, allowing *MBP* to get into the bloodstream more easily. Berger et al., found that higher

levels of *MBP* were linked to worse outcomes for children with brain damage [161]. Despite the fact that serum concentrations of *MBP* have not been evaluated in individuals who have suffered a mTBI, the potential of this protein to open the BBB [162,163] suggested that it might be measurable following mTBI.

Acid-sensing ion channel 1 (ASIC1)

Following a brain injury, the pH of brain tissue decreases, and the reduction is larger in patients who have suffered a more severe injury [155,165]. Decreased pH may increase the severity of TBI by activating acid-sensing ion channels (*ASICs*)[165]. *ASIC*s are part of the degenerin/epithelial sodium channel (DEG/ENaC) superfamily which are abundantly expressed in the central nervous system [166]. Because *ASIC*s are specifically gated by the proton, changes in the proton and lower extracellular pH during brain injury have an impact on ASICs channel activation [167,168]. Recent studies indicate that ASIC1 has been linked to synaptic plasticity, learning, memory, and fear response [168].

References

112. Ariza M, Pueyo R, Matarín MDM, Junqué C, Mataró M, Clemente I, Moral P, Poca MA, Garnacho Á, Sahuquillo J. Influence of APOE polymorphism on cognitive and behavioural outcome in moderate and severe traumatic brain injury. J Neurol Neurosurg Psychiatry . 2006;77:1191. doi: 10.1136/JNNP.2005.085167. Cited in: PMID: 16614010.

113. Nathan BP, Bellosta S, Sanan DA, Weisgraber KH, Mahley RW, Pitas RE. Differential Effects of Apolipoproteins E3 and E4 on Neuronal Growth in Vitro. Science. 1994;264:850–852. doi: 10.1126/science.8171342. Cited in: PMID: 8171342.

114. Veinbergs I, Mante M, Jung MW, van Uden E, Masliah E. Synaptotagmin and synaptic transmission alterations in apolipoprotein E-deficient mice. Prog Neuropsychopharmacol Biol Psychiatry. 1999;23:519–531. doi: 10.1016/S0278-5846(99)00013-5. Cited in: PMID: 10378234.

115. MAHLEY RW, NATHAN BP, PITAS RE. Apolipoprotein E. Structure, Function, and Possible Roles in Alzheimer's Diseasea. Ann N Y Acad Sci. 1996;777:139–145. doi: 10.1111/j.1749-6632.1996.tb34412.x. Cited in: PMID: 8624076.

116. Lawrence DW, Comper P, Hutchison MG, Sharma B. The role of apolipoprotein E episilon (ε)-4 allele on outcome following traumatic brain injury: A systematic review. https://doi.org/103109/0269905220151005131. 2015;29:1018–1031. doi: 10.3109/02699052.2015.1005131. Cited in : PMID: 25915580.

117. Chiang MF, Chang JG, Hu CJ, Dunn L, Nicoll JAR. Association between apolipoprotein E genotype and outcome of traumatic brain injury. Acta Neurochirurgica. 2003;145:649–654. doi: 10.1007/S00701-003-0069-3. Cited in : PMID: 14520543.

118. Adibhatla RM, Hatcher JF. Role Of Lipids In Brain Injury And Diseases. Future Lipidol. 2007;2:403–422. doi: 10.2217/17460875.2.4.403. Cited in: PMID: 18176634.

24. Padgett CR, Summers MJ, Vickers JC, McCormack GH, Skilbeck CE. Exploring the effect of the apolipoprotein E (APOE) gene on executive function, working memory, and processing speed during the early recovery period following traumatic brain injury. 2016;38:551–560. doi: 10.1080/13803395.2015.1137557. Cited in : PMID: 26898659.

119. Pelinka LE, Kroepfl A, Leixnering M, Buchinger W, Raabe A, Redl H. GFAP Versus S100B in Serum after Traumatic Brain Injury: Relationship to Brain Damage and Outcome. J Neurotrauma. 2004;21:1553–1561. doi: 10.1089/neu.2004.21.1553. Cited in: PMID: 15684648.

105. Metting Z, Wilczak N, Rodiger LA, Schaaf JM, van der Naalt J. GFAP and S100B in the acute phase of mild traumatic brain injury. Neurology. 2012;78:1428–1433. doi: 10.1212/WNL.0B013E318253D5C7. Cited in: PMID: 22517109.

23. Papa L, Brophy GM, Welch RD, Lewis LM, Braga CF, Tan CN, Ameli NJ, Lopez MA, Haeussler CA, Mendez Giordano DI, et al. Time Course and Diagnostic Accuracy of Glial and Neuronal Blood Biomarkers GFAP and UCH-L1 in a Large Cohort of Trauma Patients With and Without Mild Traumatic Brain Injury. JAMA Neurol. 2016;73:551–560. doi: 10.1001/JAMANEUROL.2016.0039. Cited in : PMID: 27018834.

120. Ribotta MG, Menet V, Privat A. Glial scar and axonal regeneration in the CNS: lessons from GFAP and vimentin transgenic mice. Mechanisms of Secondary Brain Damage from Trauma and Ischemia. Vienna: Springer Vienna. 2004;89:87-92. doi: 10.1007/978-3-7091-0603-7_12. Cited in: PMID: 15335106.

121. Honda M, Tsuruta R, Kaneko T, Kasaoka S, Yagi T, Todani M, Fujita M, Izumi T, Maekawa T. Serum Glial Fibrillary Acidic Protein Is a Highly Specific Biomarker for Traumatic Brain Injury in Humans Compared With S-100B and Neuron-Specific Enolase. Journal of Trauma: Injury, Infection & Critical Care. 2010;69:104–109. doi: 10.1097/TA.0b013e3181bbd485. Cited in: PMID: 20093985.

122. Kim HJ, Tsao JW, Stanfill AG. The current state of biomarkers of mild traumatic brain injury. JCI Insight. 201811;3:e97105. doi: 10.1172/jci.insight.97105. Cited in: PMID: 29321373

123. Pelinka LE, Kroepfl A, Schmidhammer R, Krenn M, Buchinger W, Redl H, Raabe A. Glial Fibrillary Acidic Protein in Serum After Traumatic Brain Injury and Multiple Trauma. The Journal of Trauma: Injury, Infection, and Critical Care. 2004;57:1006–1012. doi: 10.1097/01.TA.0000108998.48026.C3. Cited in: PMID: 15580024.

124. Huang EJ, Reichardt LF. Neurotrophins: Roles in Neuronal Development and Function. Annu Rev Neurosci. 2001;24:677. doi: 10.1146/ANNUREV.NEURO.24.1.677. Cited in: PMID: 11520916.

27. Gustafsson D, Klang A, Thams S, Rostami E. The Role of BDNF in Experimental and Clinical Traumatic Brain Injury. Int J Mol Sci. 2021;22:3582. doi: 10.3390/ijms22073582. Cited in: PMID: 33808272

125. LIPSKY RH, MARINI AM. Brain-Derived Neurotrophic Factor in Neuronal Survival and Behavior-Related Plasticity. Ann N Y Acad Sci. 2007;1122:130–143. doi: 10.1196/annals.1403.009. Cited in: PMID: 18077569.

126. Lyons WE, Mamounas LA, Ricaurte GA, Coppola V, Reid SW, Bora SH, Wihler C, Koliatsos VE, Tessarollo L. Brain-derived neurotrophic factor-deficient mice develop aggressiveness and hyperphagia in conjunction with brain serotonergic abnormalities. Proceedings of the National Academy of Sciences. 1999;96:15239–15244. doi: 10.1073/pnas.96.26.15239. Cited in: PMID: 10611369

127. Egan MF, Kojima M, Callicott JH, Goldberg TE, Kolachana BS, Bertolino A, Zaitsev E, Gold B, Goldman D, Dean M, et al. The BDNF val66met Polymorphism Affects Activity-Dependent Secretion of BDNF and Human Memory and Hippocampal Function. Cell. 2003;112:257–269. doi: 10.1016/S0092-8674(03)00035-7. Cited in: PMID: 12553913.

128. Persson L, Hårdemark HG, Gustafsson J, Rundström G, Mendel-Hartvig I, Esscher T, Påhlman S. S-100 protein and neuron-specific enolase in cerebrospinal fluid and serum: markers of cell damage in human central nervous system. Stroke. 1987;18:911–918. doi: 10.1161/01.STR.18.5.911. Cited in: PMID: 3629651.

129. Olsson B, Zetterberg H, Hampel H, Blennow K. Biomarker-based dissection of neurodegenerative diseases. Prog Neurobiol. 2011;95:520–534. doi: 10.1016/j.pneurobio.2011.04.006. Cited in: PMID: 21524681.

130. Kempuraj D. Current Trends in Biomarkers for Traumatic Brain Injury. Open Access J Neurol Neurosurg. 2020;12. doi: 10.19080/OAJNN.2020.12.555842.

131. Blyth BJ, Farahvar A, He H, Nayak A, Yang C, Shaw G, Bazarian JJ. Elevated Serum Ubiquitin Carboxy-Terminal Hydrolase L1 Is Associated with Abnormal Blood–Brain Barrier Function after Traumatic Brain Injury. J Neurotrauma. 2011;28:2453–2462. doi: 10.1089/neu.2010.1653. Cited in: PMID: 21428722.

22. Thelin EP, Johannesson L, Nelson D, Bellander BM. S100B Is an Important Outcome Predictor in Traumatic Brain Injury. https://home.liebertpub.com/neu. 2013;1;30:519–528. doi: 10.1089/NEU.2012.2553. Cited in: PMID: 23297751.

132. Witte AV, Flöel A. Effects of COMT polymorphisms on brain function and behavior in health and disease. Brain Res Bull. 2012;88:418–428. doi: 10.1016/J.BRAINRESBULL.2011.11.012. Cited in: PMID: 22138198.

107. Lipsky RH, Sparling MB, Ryan LM, Xu K, Salazar AM, Goldman D, Warden DL. Association of COMT Val158Met Genotype With Executive Functioning Following Traumatic Brain Injury. J Neuropsychiatry Clin Neurosci. 2005;17:465–471. doi: 10.1176/jnp.17.4.465. Cited in: PMID: 16387984.

81. Failla MD, Myrga JM, Ricker JH, Edward Dixon C, Conley YP, Wagner AK. Post-TBI cognitive performance is moderated by variation within ANKK1 and DRD2 genes. J Head Trauma Rehabil. 2015;30:E54. doi: 10.1097/HTR.0000000000000118. Cited in: PMID: 25931179.

133. Braver TS, Cohen JD. On the Control of Control: The Role of Dopamine in Regulating Prefrontal Function and Working Memory.

134. Aalto S, Brück A, Laine M, Någren K, Rinne JO. Frontal and Temporal Dopamine Release during Working Memory and Attention Tasks in Healthy Humans: a Positron Emission Tomography Study Using the High-Affinity Dopamine D2 Receptor Ligand [11C]FLB 457. The Journal of Neuroscience. 2005;25:2471. doi: 10.1523/JNEUROSCI.2097-04.2005. Cited in : PMID: 15758155.

80. Bales JW, Wagner AK, Kline AE, Dixon CE. Persistent cognitive dysfunction after traumatic brain injury: A dopamine hypothesis. Neurosci Biobehav Rev. 2009;33:981–1003. doi: 10.1016/J.NEUBIOREV.2009.03.011. Cited in: PMID: 19580914.

135. McAllister TW, Rhodes CH, Flashman LA, McDonald BC, Belloni D, Saykin AJ. Effect of the dopamine D2 receptor T allele on response latency after mild traumatic brain injury. Am J Psychiatry. 2005;162:1749–1751. doi: 10.1176/APPI.AJP.162.9.1749. Cited in: PMID: 16135640.

136. McAllister TW, Flashman LA, Harker Rhodes C, Tyler AL, Moore JH, Saykin AJ, McDonald BC, Tosteson TD, Tsongalis GJ. Single nucleotide polymorphisms in ANKK1 and the dopamine D2 receptor gene affect cognitive outcome shortly after traumatic brain injury: A replication and extension study. Brain Inj. 2008;22:705–714. doi: 10.1080/02699050802263019. Cited in: PMID: 18698520.

137. Elwood RW. The California Verbal Learning Test: Psychometric characteristics and clinical application. Neuropsychol Rev. 1995;5:173–201. doi: 10.1007/BF02214761. Cited in: PMID: 8653108.

138. Kors EE, Terwindt GM, Vermeulen FLMG, Fitzsimons RB, Jardine PE, Heywood P, Love S, van den Maagdenberg AMJM, Haan J, Frants RR, et al. Delayed cerebral edema and fatal coma after minor head trauma: Role of the CACNA1A calcium channel subunit gene and relationship with familial hemiplegic migraine. Ann Neurol. 2001;49:753–760. doi: 10.1002/ana.1031. Cited in: PMID: 8653108.

139. McAllister TW. Genetic factors in traumatic brain injury. Handb Clin Neurol. 2015;128:723-39. doi: 10.1016/B978-0-444-63521-1.00045-5. Cited in: PMID: 25701917.

140. Stam AH, Luijckx G-J, Poll-The BT, Ginjaar IB, Frants RR, Haan J, Ferrari MD, Terwindt GM, van den Maagdenberg AMJM. Early seizures and cerebral oedema after trivial head trauma associated with the CACNA1A S218L mutation. J Neurol Neurosurg Psychiatry. 2009;80:1125–1129. doi: 10.1136/jnnp.2009.177279. Cited in: PMID: 19520699.

141. Dardiotis E, Paterakis K, Tsivgoulis G, Tsintou M, Hadjigeorgiou GF, Dardioti M, Grigoriadis S, Simeonidou C, Komnos A, Kapsalaki E, et al. AQP4 Tag Single Nucleotide Polymorphisms in Patients with Traumatic Brain Injury. J Neurotrauma. 2014;31:1920–1926. doi: 10.1089/neu.2014.3347. Cited in: PMID: 24999750

142. Badaut J, Lasbennes F, Magistretti PJ, Regli L. Aquaporins in Brain: Distribution, Physiology, and Pathophysiology. Journal of Cerebral Blood Flow & Metabolism. 2002;22:367–378. doi: 10.1097/00004647-200204000-00001. Cited in: PMID: 11919508

143. Aoki K, Uchihara T, Tsuchiya K, Nakamura A, Ikeda K, Wakayama Y. Enhanced expression of aquaporin 4 in human brain with infarction. Acta Neuropathol. 2003;106:121–124. doi: 10.1007/S00401-003-0709-Y/FIGURES/2. Cited in: PMID: 12715185.

144. Papadopoulos MC, Verkman AS. Potential utility of aquaporin modulators for therapy of brain disorders. Prog Brain Res. 2008;170:589–601. doi: 10.1016/S0079-6123(08)00446-9. Cited in: PMID: 18655912.

145. Sorani MD, Zador Z, Hurowitz E, Yan D, Giacomini KM, Manley GT. Novel variants in human Aquaporin-4 reduce cellular water permeability. Hum Mol Genet. 2008;17:2379–2389. doi: 10.1093/hmg/ddn138.

146. Donkin JJ, Vink R. Mechanisms of cerebral edema in traumatic brain injury: Therapeutic developments. Curr Opin Neurol. 2010;23:293–299. doi: 10.1097/WCO.0B013E328337F451. Cited in: PMID: 20168229.

147. Ghabriel MN, Thomas A, Vink R. Magnesium restores altered aquaporin-4 immunoreactivity following traumatic brain injury to a pre-injury state. Acta Neurochir Suppl (Wien). 2006;96:402–406. doi: 10.1007/3-211-30714-1_83/COVER. Cited in: PMID: 16671494.

104. Day INM, Thompson RJ. UCHL1 (PGP 9.5): neuronal biomarker and ubiquitin system protein. Prog Neurobiol. 2010;90:327–362. doi: 10.1016/J.PNEUROBIO.2009.10.020. Cited in: PMID: 19879917.

148. Shahjouei S, Sadeghi-Naini M, Yang Z, Kobeissy F, Rathore D, Shokraneh F, Blackburn S, Manley GT, Wang KKW. The diagnostic values of UCH-L1 in traumatic brain injury: A meta-analysis. https://doi.org/101080/0269905220171382717. 2017;32:1–17. doi: 10.1080/02699052.2017.1382717. Cited in: PMID: 29087740.

149. Mondello S, Shear DA, Bramlett HM, Dixon CE, Schmid KE, Dietrich WD, Wang KKW, Hayes RL, Glushakova O, Catania M, et al. Insight into Pre-Clinical Models of Traumatic Brain Injury Using Circulating Brain Damage Biomarkers: Operation Brain Trauma Therapy. J Neurotrauma. 2016;33:595–605. doi: 10.1089/neu.2015.4132. Cited in: PMID: 26671651.

150. Moss A, Blackburn-Munro G, Garry EM, Blakemore JA, Dickinson T, Rosie R, Mitchell R, Fleetwood-Walker SM. A role of the ubiquitin-proteasome system in neuropathic pain. J Neurosci. 2002;22:1363–1372. doi: 10.1523/JNEUROSCI.22-04-01363.2002. Cited in: PMID: 11850463.

151. Setsuie R, Wada K. The functions of UCH-L1 and its relation to neurodegenerative diseases. Neurochem Int. 2007;51:105–111. doi: 10.1016/j.neuint.2007.05.007. Cited in: PMID: 17586089

152. Bishop P, Rocca D, Henley JM. Ubiquitin C-terminal hydrolase L1 (UCH-L1): structure, distribution and roles in brain function and dysfunction. Biochemical Journal. 2016;473:2453–2462. doi: 10.1042/BCJ20160082. Cited in: PMID: 27515257

153. Jackson P, Thompson RJ. The demonstration of new human brain-specific proteins by high-resolution two-dimensional polyacrylamide gel electrophoresis. J Neurol Sci. 1981;49:429–438. doi: 10.1016/0022-510X(81)90032-0. Cited in: PMID: 7217993.

154. Todi S v., Paulson HL. Balancing act: deubiquitinating enzymes in the nervous system. Trends Neurosci. 2011;34:370–382. doi: 10.1016/j.tins.2011.05.004. Cited in: PMID: 21704388

155. Liu MC, Akinyi L, Scharf D, Mo J, Larner SF, Muller U, Oli MW, Zheng W, Kobeissy F, Papa L, et al. Ubiquitin C-terminal hydrolase-L1 as a biomarker for ischemic and traumatic brain injury in rats. European Journal of Neuroscience. 2010;31:722–732. doi: 10.1111/j.1460-9568.2010.07097.x. Cited in: PMID: 20384815

156. Rafter D, Li Z, Schaaf T, Gault K, Thorpe M, Venkatesh S, Edpuganti R, Song T, Kuang R, Samadani U, et al. Machine Learning with Objective Serum Markers and Algorithmic Deep Learning Computed Tomography Scan Analysis for Classification of Brain Injury. medRxiv. 2021;2021.02.13.21250776. doi: 10.1101/2021.02.13.21250776.

157. Papa L, Akinyi L, Liu MC, Pineda JA, Tepas JJ, Oli MW, Zheng W, Robinson G, Robicsek SA, Gabrielli A, et al. Ubiquitin C-terminal hydrolase is a novel biomarker in humans for severe traumatic brain injury*. Crit Care Med. 2010;38:138–144. doi: 10.1097/CCM.0b013e3181b788ab. Cited in: PMID: 19726976

158. Barbarese E, Barry C, Chou CJ, Goldstein DJ, Nakos GA, Hyde-DeRuyscher R, Scheld K, Carson JH. Expression and Localization of Myelin Basic Protein in Oligodendrocytes and Transfected Fibroblasts. J Neurochem . 1988;51:1737–1745. doi: 10.1111/J.1471-4159.1988.TB01153.X. Cited in: PMID: 2460587.

159. Berger RP, Adelson PD, Pierce MC, Dulani T, Cassidy LD, Kochanek PM. Serum neuron-specific enolase, S100B, and myelin basic protein concentrations after inflicted and noninflicted traumatic brain injury in children. J Neurosurg Pediatr. 2005;103:61–68. doi: 10.3171/PED.2005.103.1.0061. Cited in: PMID: 16122007.

160. Kochanek PM, Berger RP, Bayr H, Wagner AK, Jenkins LW, Clark RS. Biomarkers of primary and evolving damage in traumatic and ischemic brain injury: diagnosis, prognosis, probing mechanisms, and therapeutic decision making. Curr Opin Crit Care. 2008;14:135–141. doi: 10.1097/MCC.0b013e3282f57564. Cited in: PMID: 18388674

161. Berger RP, Bazaco MC, Wagner AK, Kochanek PM, Fabio A. Trajectory Analysis of Serum Biomarker Concentrations Facilitates Outcome Prediction after Pediatric Traumatic and Hypoxemic Brain Injury. Dev Neurosci. 2010;32:396–405. doi: 10.1159/000316803. Cited in: PMID: 20847541

162. D'Aversa TG, Eugenin EA, Lopez L, Berman JW. Myelin basic protein induces inflammatory mediators from primary human endothelial cells and blood-brain barrier disruption: implications for the pathogenesis of multiple sclerosis. Neuropathol Appl Neurobiol. 2013;39:270–283. doi: 10.1111/J.1365-2990.2012.01279.X. Cited in: PMID: 22524708.

163. Jeter CB, Hergenroeder GW, Hylin MJ, Redell JB, Moore AN, Dash PK. Biomarkers for the Diagnosis and Prognosis of Mild Traumatic Brain Injury/Concussion. J Neurotrauma. 2013;30:657–670. doi: 10.1089/neu.2012.2439. Cited in: PMID: 23062081

164. Clausen T, Khaldi A, Zauner A, Reinert M, Doppenberg E, Menzel M, Soukup J, Alves OL, Bullock MR. Cerebral acid—base homeostasis after severe traumatic brain injury. J Neurosurg. 2005;103:597–607. doi: 10.3171/jns.2005.103.4.0597. Cited in: PMID: 16266040

165. Yin T, Lindley TE, Albert GW, Ahmed R, Schmeiser PB, Grady MS, Howard MA, Welsh MJ. Loss of Acid Sensing Ion Channel-1a and Bicarbonate Administration Attenuate the Severity of Traumatic Brain Injury. PLoS One. 2013;8:e72379. doi: 10.1371/journal.pone.0072379. Cited in: PMID: 23991103

166. Wemmie JA, Chen J, Askwith CC, Hruska-Hageman AM, Price MP, Nolan BC, Yoder PG, Lamani E, Hoshi T, Freeman JH, et al. The Acid-Activated Ion Channel ASIC Contributes to Synaptic Plasticity,

Learning, and Memory. Neuron. 2002;34:463–477. doi: 10.1016/S0896-6273(02)00661-X. Cited in: PMID: 11988176.

167. Xiong ZG, Chu XP, Simon RP. Acid sensing ion channels - Novel therapeutic targets for ischemic brain injury. Frontiers in Bioscience. 2007;12:1376–1386. doi: 10.2741/2154/PDF. Cited in: PMID: 33550975

168. Wemmie JA, Askwith CC, Lamani E, Cassell MD, Freeman JH, Welsh MJ. Acid-Sensing Ion Channel 1 Is Localized in Brain Regions with High Synaptic Density and Contributes to Fear Conditioning. The Journal of Neuroscience. 2003;23:5496–5502. doi: 10.1523/JNEUROSCI.23-13-05496.2003. Cited in: PMID: 12843249
